# Perspective improvement of regional air pollution burden of disease estimation by machine intelligence

**DOI:** 10.3389/fpubh.2025.1436838

**Published:** 2025-03-12

**Authors:** Cheng-Pin Kuo, Joshua S. Fu, Yang Liu

**Affiliations:** ^1^Industrial Technology Research Institute, Hsinchu, Taiwan; ^2^Department of Civil and Environmental Engineering, University of Tennessee Knoxville, Knoxville, TN, United States; ^3^Gangarosa Department of Environmental Health, Emory University, Atlanta, GA, United States

**Keywords:** PM_2.5_, ozone, machine learning, disease burden, bias correction

## Abstract

As air pollution events increasingly threaten public health under climate change, more precise estimations of air pollutant exposure and the burden of diseases (BD) are urgently needed. However, current BD assessments from various sources of air pollutant concentrations and exposure risks, and the derived uncertainty still needs systematic assessment. Owing to growing health and air quality data availability, machine learning (ML) may provide a promising solution. This study proposed an ML-measurement-model fusion (MMF) framework that can quantify the air pollutant biases from the Chemical Transport Modeling (CTM) inputs, and further analyze the BD biases concerning various sources of air pollutant estimations and exposure risks. In our study region, the proposed ML-MMF framework successfully improves CTM-modeled PM_2.5_ (from R^2^ = 0.41 to R^2^ = 0.86) and O_3_ (from R^2^ = 0.48 to R^2^ = 0.82). The bias quantification results showed that premature deaths in the study region are mainly biased by boundary conditions (Improvement Ratio, IR = 99%) and meteorology (91%), compared with emission and land-use data. The results of further analysis showed using observations only (PM_2.5_: 17%; O_3_: 56%) or the uncorrected CTM estimations (PM_2.5_: −18%; O_3_: 171%) contributed more BD biases compared with employing averaged risks without considering urbanization levels (PM_2.5_: −5%; O_3_: −4%). In conclusion, employing observations only, uncorrected CTM estimations, and homogeneous risks may contribute to non-negligible BD biases and affect regional air quality and risk management. To cope with increasing needs of finer-scale air quality management under climate change, our developed ML-MMF framework can provide a quantitative reference to improve CTM performance and priority to improve input data quality and CTM mechanisms.

## 1 Introduction

The burden of the disease (BD) has been extensively employed to describe the impact of exposure to ambient air pollution on regional and global air quality and risk management. The Global Burden of Disease (GBD) project estimated that about 2.94 and 0.47 million premature deaths worldwide could be attributed to ambient particulate matter and ozone (O_3_) pollution, respectively (1). However, due to the assumptions of the Integrated Exposure-Response (IER) algorithm employed by GBD, the GBD estimations more focus on long-term and cumulative exposure but overlook the temporal fluctuation of short-term exposure (2). Since extreme events such as wildfires and transboundary pollution have frequently deteriorated regional air quality (3, 4), short-term air pollution exposure and the derived acute BDs should be further studied and systematically investigated.

The current BD estimations remain significant uncertainty due to various sources of air pollutant concentrations and exposure risks. PM_2.5_- and/or O_3_-derived BD estimations usually rely on air pollutant observations and/or Chemical Transport Model (CTM) estimations and city-level or nation-specific exposure risks. When local health and air quality data in regions, cities, and communities become more available, finer-scale air quality and risk management have gradually come to be recognized, and hourly or daily PM_2.5_ and O_3_ exposure estimations are urgently needed, but the exploration of exposure uncertainty from either observations or CTM still have limited improvements and remain discrepant.

CTMs have been applied to simulate air pollutant concentrations for decades due to their capability to model air quality for areas without observations. Their numerical algorithms and knowledge-based inputs also facilitate users to explore environmental issues and predict future trends (5, 6). However, current estimations from CTMs or ensemble databases such as the global chemistry transport model (GEOS-Chem), Community Multiscale Air Quality Modeling System (CMAQ), or the Model Inter-Comparison Study for Asia (MICS- Asia) (6) were directly verified by limited observations and remained significant bias. Demanding computational resources and time also slows down model improvement (7, 8).

Owing to the development of environmental monitoring techniques and increasingly available environmental data in recent years, machine learning (ML) has provided effective and promising applications to improve the accuracy of CTM predictions (9–11). ML algorithms such as regression-based model (12), tree-based model (13), and neural networks (14, 15) have been utilized to correct modeling results and develop Measurement-model fusion (MMF) techniques based on observations, emission data, meteorological data, land-use data or other auxiliary data (16). For example, Lu et al. (15) employed three ML methods coupling with the CMAQ model to forecast O_3_ concentration, and the results showed that long short-term memory recurrent neural network (LSTM-RNN) can reduce most biases and had the best performance among three ML methods (15). Sayeed et al. (17) used meteorological data, CMAQ outputs, and observations and applied a convolutional neural network (CNN) to forecast air pollutants such as PM_2.5_, PM_10_, and NO_2_, and the CNN model improved the yearly index of the agreement by 13–40% for the selected pollutants (18). However, although previous studies proved the capabilities of ML and DL techniques to improve modeling performance, the biases between modeled estimations and observations were not systematically investigated.

Technically, the bias of CTM estimation is the difference between modeling estimation and observation and affected by modeling inputs including emission inventory, boundary conditions, local meteorology, and land-use data (15, 18). Multiple reasons such as inaccurate modeling inputs (19, 20), accumulation of input biases during the modeling process, and imperfect chemical and physical mechanisms (21) in the model contribute to biases. However, few studies further quantified the potential confounders or input components that cause biases and derived biases in BD calculations. CTMs and modeling inputs also remained unfixed and hardly benefited from ML modeling except for corrected estimations. Moreover, although ML models showed good capabilities in bias correction, the biases between modeled estimations and observations were not systematically investigated, and the bias originating from individual modeling inputs was neither quantified.

Another potential concern of regional BD or GBD is overlooking heterogeneous exposure risks among different urbanization levels. For instance, higher premature death risks in rural areas and higher cardiovascular disease risks in urban areas due to PM_2.5_ exposure have been identified (22–24). Population density and distribution also significantly affect regional BDs estimations, which could be seriously underestimated if exposure risks in urban areas are higher than average. As most current BD calculations still employed regional or nation-level risk and population, further assessment considering risk spatial heterogeneity should be evaluated to support community-level air quality management.

To meet the needs of more accurate PM_2.5_ and O_3_ exposure assessment, improvement of CTM modeling performance, and finer-scale air quality and risk management, this study proposed the machine learning-measurement-model fusion (ML-MMF) framework that can improve CTM modeling performance, quantify the sources of CTM estimation biases from the modeling inputs and further explore the bias of BD derived from different PM_2.5_ and O_3_ concentration data, and exposure risks. Taiwan was selected as the study region due to its isolated geography, well-established air quality monitoring network, routinely updated emission inventory, and available health insurance database. The goal of this study is to improve PM_2.5_/O_3_ concentrations and premature death estimations, quantify the sources of CTM estimation biases from the modeling inputs (emissions, boundary conditions, local meteorology, and land uses), and compare premature deaths from different PM_2.5_/O_3_ concentration data and exposure risks. Section 2 introduces dataset preparation (Section 2.1), the developed ML-MMF framework in this study, the bias quantification techniques (Section 2.2), and the use of the burden of the disease estimation for sensitivity analysis (Section 2.3). Section 3 illustrates the improved performance of modeling results (Section 3.1), the results of PM_2.5_ and O_3_ modeling bias quantification (Section 3.2), and the results of further analysis considering different parameters to calculate the burden of the disease (Section 3.3). Section 4 elaborates on the proposed perspectives (Section 4.1) and limitations (Section 4.2) of this study.

## 2 Methodology

### 2.1 Dataset preparation

The weather research and forecasting model (WRF, version 3.8) and CMAQ model (version 5.2) with the Carbon Bond 6 and AERO6 mechanisms were used to simulate meteorological fields and air pollutant concentrations, respectively. The WRF-CMAQ modeling nested four layers from East Asia (81 km × 81 km) to Taiwan island (3 km × 3 km) which covers 90 (row) × 135 (column) horizontal grid cells (Supplementary Figure S1) (25, 26). Emissions were from the Taiwan Emission Data System (TEDS) version 10.0 which was developed by the Taiwan Environmental Protection Administration (Taiwan EPA).

**Figure 1:**
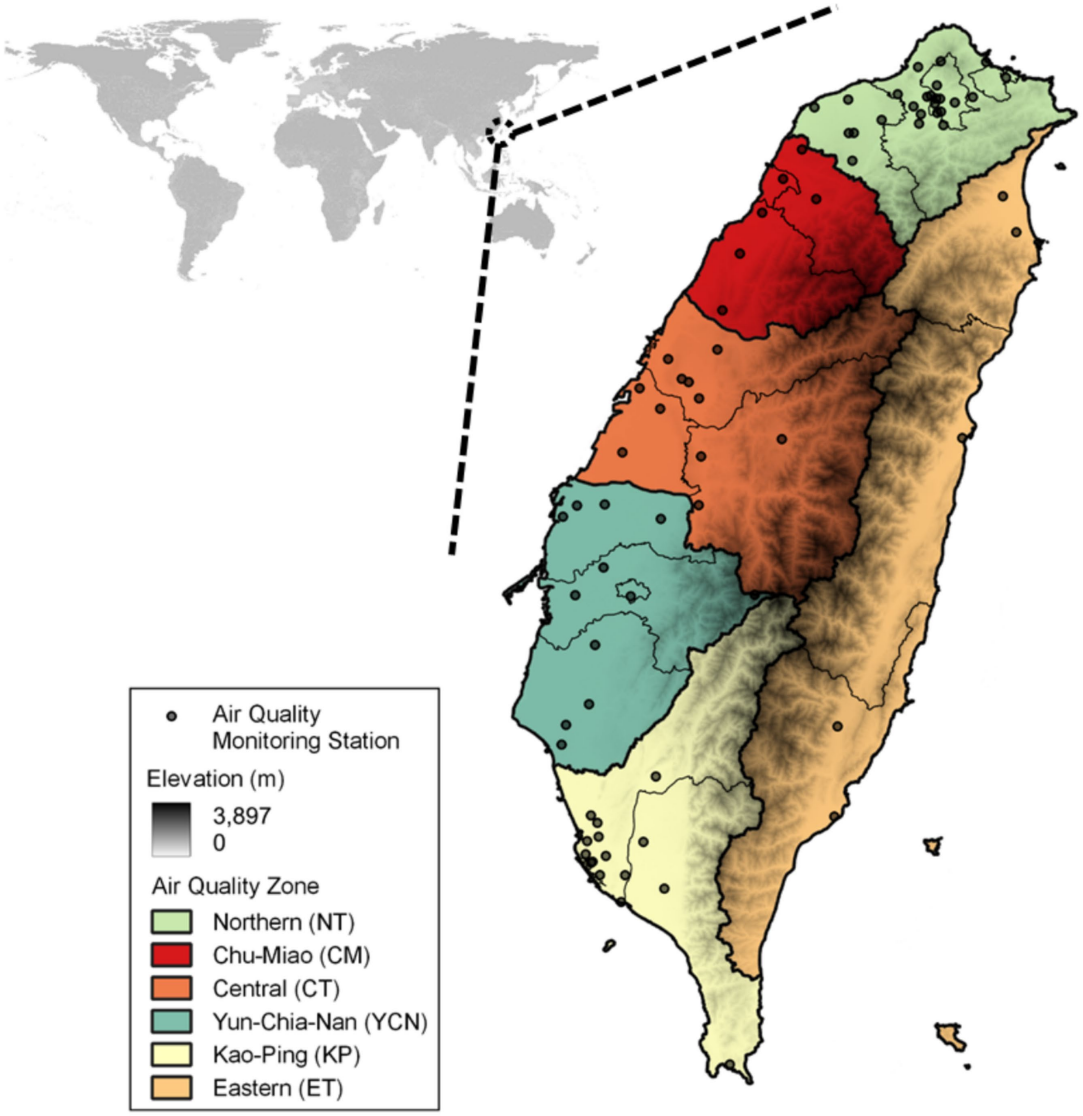
Air quality monitoring network (*n* = 73) and air quality regions in Taiwan.

ML-MMF input variables are retrieved from CMAQ inputs including emissions, boundary conditions, meteorology, and land-use data (Supplementary Table S1). Hourly observational data of PM_2.5_ and O_3_ in January, April, July, and October 2016 from 73 air quality monitoring stations (Figure 1) in six air quality regions were used. Daily PM_2.5_ and maximum daily 8-h ozone average (MDA8 O_3_) were calculated based on the standards of the World Health Organization (WHO) and Taiwan EPA and used as the dependent variables. The chosen independent variables are related to the emission of precursors (PM_2.5_, NOx, SOx, NH3, and VOCs) and meteorological conditions. Meteorological factors on 850 and 690 hPa layers were selected to represent the weather conditions of the mixing layer and low troposphere layer (15).

### 2.2 ML-MMF framework

**Figure 2:**
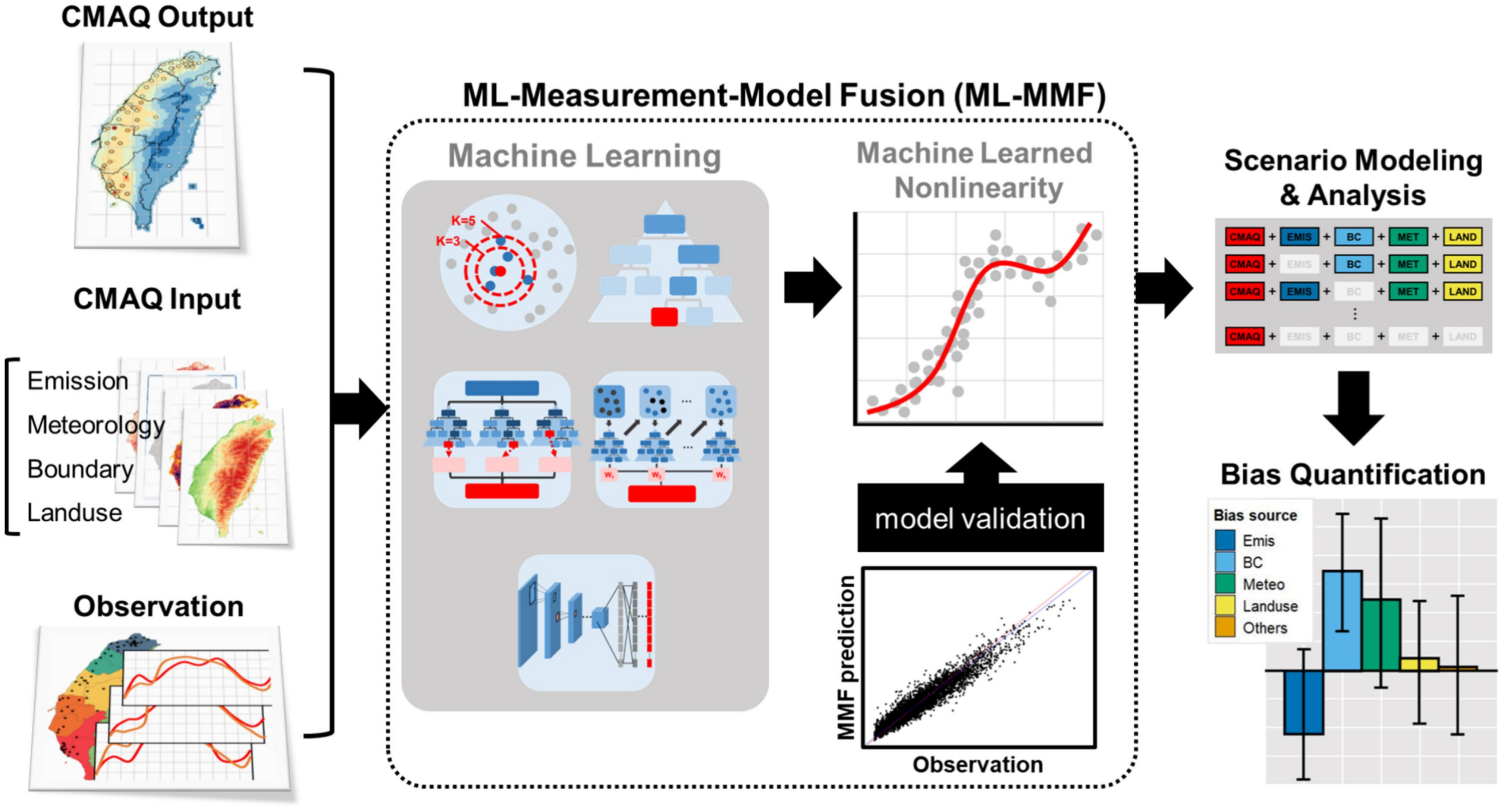
Technical flowchart of the proposed machine learning-measurement model fusion (ML-MMF) framework for PM_2.5_ and O_3_ prediction.

The flowchart of the ML-MMF framework is presented in Figure 2. First, all inputs served as predictors including CMAQ output, emissions, boundary conditions, meteorology, and land-use data were aggregated to the same resolution (3 km × 3 km); Observations were further combined to predictor datasets, and the grid cells having observations were used for the learning process. A random selection was employed; 60% of the data set was selected as the training dataset, and 40% was used as the testing dataset. Second, five ML techniques including the k-nearest neighbors’ regression (KNN), regression tree (RT), random forest (RF), gradient-boosted tree models (GBM), and convolutional neural network (CNN) that can deal with non-linearity were trained with a 10-fold cross-validation to predict daily PM_2.5_ and MDA8 O_3_ with the best schemes. Finally, the testing dataset was applied to all models to validate the predictions, and the best algorithm was used for further analysis (27).

### 2.3 Bias quantification

Different scenarios were designed to quantify bias between CMAQ raw output and observations (Table 1 and Appendix I). S1_BASE is the base scenario that uses all inputs for prediction and serves as a baseline for comparing with the other scenarios, and the following scenarios illustrate individual improved performance by including each data (emission, boundary condition, meteorological, and land-use data) for MMF. The bias quantification technique utilized PM_2.5_ and O_3_ estimations from each scenario. For each region, total bias ( $\Delta C_{\mathrm{Total}}$ ) was defined by the changing population-weighted PM_2.5_ and O_3_ between CMAQ raw output and S1_BASE ( $\Delta C_{\mathrm{Total}}=C_{\mathrm{CMAQ}}-C_{S1\_\mathrm{BASE}}$ ). The modeling capability of each component was defined by the following scenarios (S2-S5). For example, the modeling capability of emissions was defined by the changing concentration between CMAQ output and S2_EM ( $\Delta C_{1,EM}=C_{\mathrm{CMAQ}}-C_{S2\_EM}$ ). For all components, the calculated biases were further used to apportion their contributions to the total bias through multiple linear regression (MLR):


$$\Delta C_{\mathrm{Total}}=\beta_{0}+\beta_{1}\Delta C_{i,EM}+\beta_{2}\Delta C_{i,BC}+\beta_{3}\Delta C_{i,MT}+\beta_{4}\Delta C_{i,\mathrm{LU}}+\varepsilon$$

where i represent the application of the scenarios ( $\Delta C_{EM}$ , $\Delta C_{BC}$ , $\Delta C_{MT}$ , and $\Delta C_{\mathrm{LU}}$ ); $\beta_{0}$ is the intercept; $\beta_{1}$ to $\beta_{4}$ represents contributed bias with a unit increase of delta PM_2.5_ or O_3_ concentration. The products including $\beta_{1}\Delta C_{EM}$ , $\beta_{2}\Delta C_{BC}$ , $\beta_{3}\Delta C_{MT}$ , and $\beta_{4}\Delta C_{\mathrm{LU}}$ are the changed concentrations from emissions, boundary conditions, meteorology, and land-use data, respectively. ε are residuals and represent biases from other unidentified factors.

**Table 1:** Modeling performance evaluation (R^2^) of PM_2.5_ and O_3_ for different ML techniques and scenarios.

Scenario	Input data	KNN		RT		RF		GBM		CNN	
		Train	Test	Train	Test	Train	Test	Train	Test	Train	Test
PM_2.5_											
S1_BASE	CMAQ+Emis+BC + Met+LU	0.68	0.68	0.76	0.77	0.87	0.87	0.86	0.86	0.95	0.83
S2_EM	CMAQ + Emis	0.51	0.54	0.47	0.48	0.59	0.59	0.58	0.59	0.62	0.57
S3_BC	CMAQ + BC	0.75	0.76	0.74	0.74	0.77	0.77	0.76	0.77	0.79	0.77
S4_MT	CMAQ + Met	0.71	0.73	0.63	0.65	0.81	0.82	0.76	0.77	0.84	0.68
S5_LU	CMAQ + LU	0.48	0.49	0.46	0.48	0.50	0.51	0.51	0.52	0.52	0.49
O_3_											
S1_BASE	CMAQ+Emis+BC + Met+LU	0.62	0.63	0.73	0.73	0.84	0.85	0.82	0.81	0.93	0.78
S2_EM	CMAQ + Emis	0.51	0.49	0.49	0.48	0.53	0.52	0.56	0.55	0.58	0.51
S3_BC	CMAQ + BC	0.78	0.77	0.75	0.74	0.78	0.78	0.77	0.77	0.79	0.76
S4_MT	CMAQ + Met	0.70	0.69	0.62	0.61	0.78	0.79	0.74	0.73	0.78	0.63
S5_LU	CMAQ + LU	0.51	0.47	0.49	0.48	0.52	0.49	0.53	0.51	0.54	0.50

### 2.4 Sensitivity analysis

Premature deaths were used to illustrate the potential BD bias by adopting different sources of air pollutant concentrations and exposure risks and are calculated from concentration-response functions (CRFs) (23, 28):


$$Y=E_{0}\cdot P\cdot \left(1-e^{-\beta \cdot \left(C-C_{0}\right)}\right)\cdot A$$

where Y is the number of premature deaths; $E_{0}$ is the mortality rate; P is the population; The coefficient β is the short-term exposure risk of acute death due to PM_2.5_ or O_3_ exposure, which would consider heterogeneous risks among different urbanization levels (Supplementary Table S2) (23); A is a scalar (1/365). $C_{0}$ is the threshold concentration. The threshold concentration was set as 25 μg/m^3^ for daily PM_2.5_ or 60 ppb for MDA8 O_3_ (29). C is the exposure concentration from observations, CMAQ, or ML-MMF estimations.

## 3 Results

### 3.1 Improved modeling performance

This section describes the improved performance of modeling results by using different data for CMAQ and ML models. The modeling performance of CMAQ and ML models for the designed scenarios is shown in Table 1. The R^2^ of CMAQ output is 0.41 and 0.48 for PM_2.5_ and O_3_, respectively, and the R^2^ of S1_BASE, including all the auxiliary data for MMF, can be enhanced to 0.68–0.95 and 0.62–0.93 for PM_2.5_ and O_3_, respectively concerning different techniques, which CNN has the highest R^2^, followed by RF and GBM.

**Figure 3:**
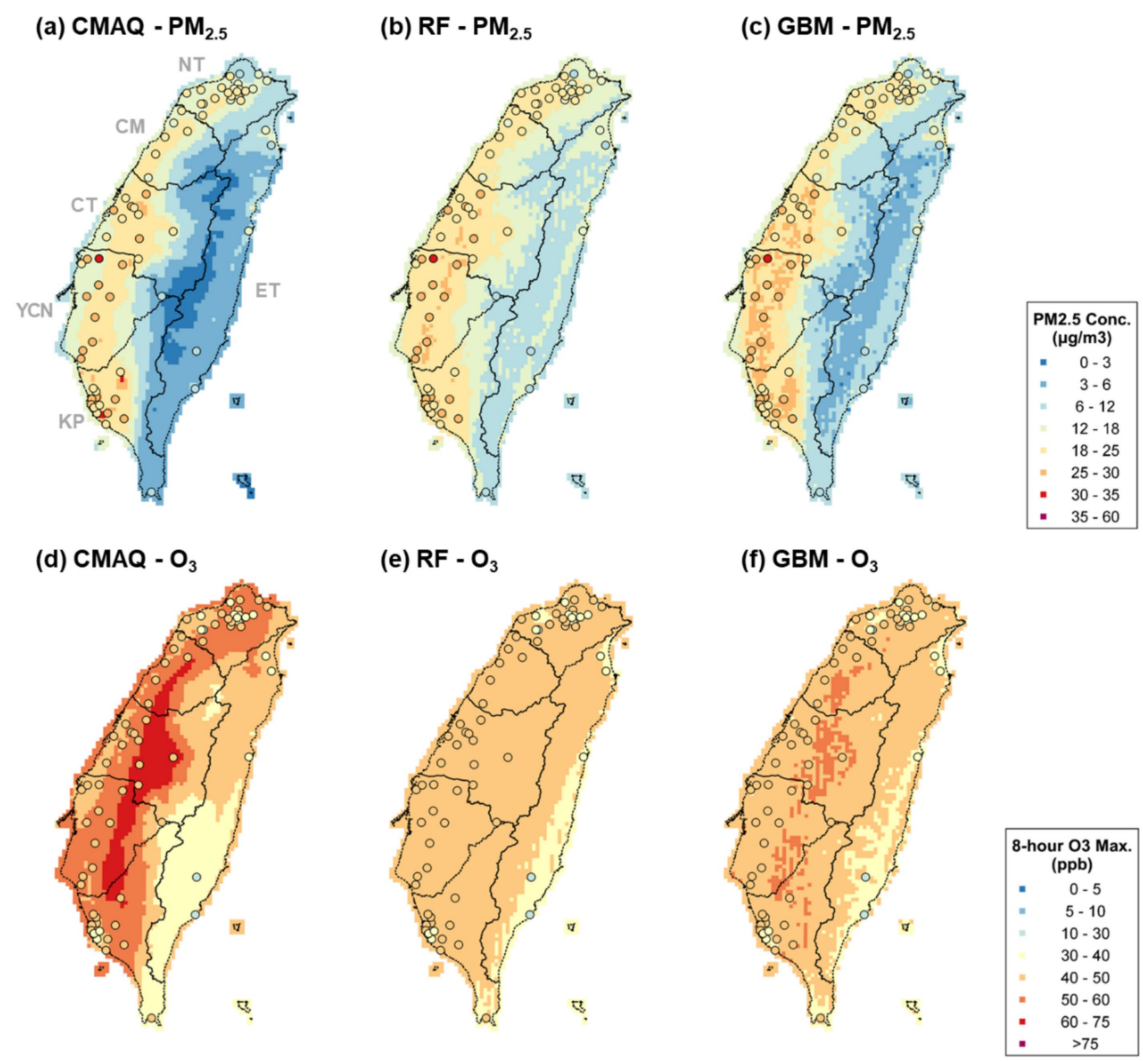
Observations (circles) and modeled estimations (grids) for PM_2.5_ **(a–c)** and O_3_ **(d–f)** from CMAQ, RF, and GBM outputs.

Considering each ML technique, however, CNN shows an overfitting tendency. Although different split portions of training and testing data were tried, the overfitting persisted. Thus, CNN results were not considered in further analysis. Next, both RF and GBM have comparable higher training R^2^ (RF: 0.87 and 0.84 for PM_2.5_ and O_3_; GBM: 0.86 and 0.82 for PM_2.5_ and O_3_). By comparing the spatial distribution between CMAQ, RF, and GBM outputs (Figure 3), PM_2.5_ and O_3_ estimations significantly approximate closer to observations after ML-MMF. CMAQ tends to underestimate PM_2.5_ and overestimate O_3_, especially in western Taiwan. By comparing CMAQ (Figures 3a,d) and GBM output (Figures 3c,f), RF (Figures 3b,e) showed relatively homogenous spatial patterns of PM_2.5_ and O_3_. For example, observed PM_2.5_ in the western area is higher than RF-modeled PM_2.5_, while GBM-modeled PM_2.5_ showed comparable estimations as observations. In addition, O_3_ accumulation at the western side of the mountains is not reflected by the RF model as well, while GBM remains the spatial patterns of O_3_ accumulation from CMAQ and observation-level estimations. The homogenous spatial patterns of RF imply its inferior performance in spatial modeling and could be due to its lower variable importance priorities of elevation and land-use characteristics, where stiff terrain slopes in Taiwan could have much impact on air pollutant concentrations. On the other hand, GBM presents a more reasonable spatial distribution of PM_2.5_ and O_3_ which are closer to observations. The significantly lower concentrations in the central mountains and the eastern valley and the higher concentrations in the western plain are elaborated by GBM. Besides, the Multiple-model (MM) ensemble approach (30) was also assessed by using RF and GBM, but the R^2^ of the ensemble approach showed significant overfitting (Supplementary Table S3). Thus, based on modeling performance, spatial evaluation, and parsimonious principle, GBM was selected, and its results were used for further analysis.

Considering the impact from individual components (Table 1), the R^2^ suggests adding boundary conditions (S3_BC, 0.77 for PM_2.5_ and 0.77 for O_3_) and meteorological factors (S4_MT, 0.77 for PM_2.5_ and 0.73 for O_3_) would largely increase ML-MMF modeling performance compared with CMAQ output (0.41 for PM_2.5_ and 0.48 for O_3_), implying that boundary conditions and meteorology contribute to most explained variance for ML-MMF.

### 3.2 Bias quantification

**Figure 4:**
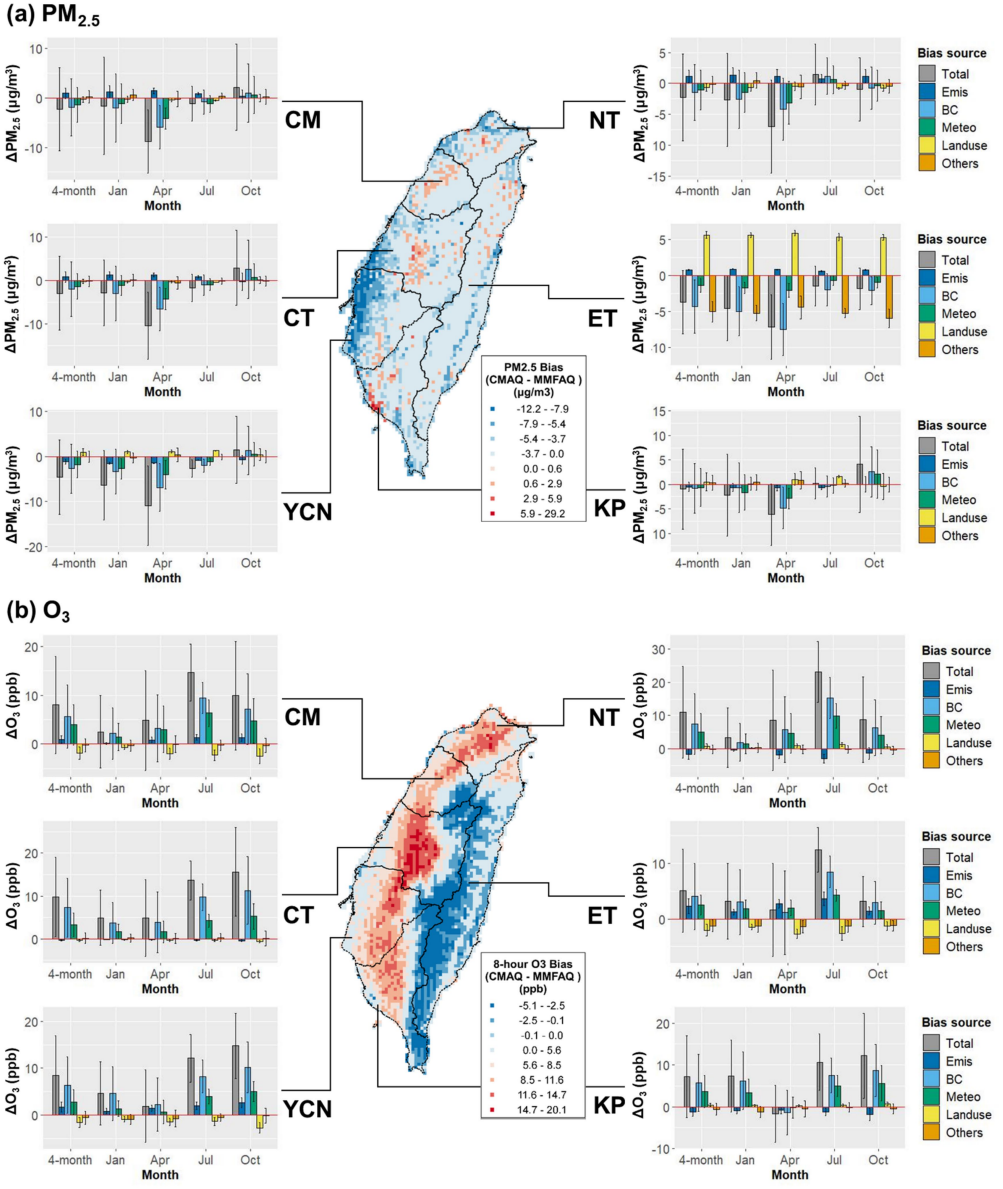
Spatial distributions of **(a)** daily PM_2.5_ and **(b)** MDA8 O_3_ maximum estimation biases and their quantified biases from emissions, boundary conditions, local meteorology, land-use data, and other unidentified factors. The total bias is defined by the subtraction of ML-MMF estimations from CMAQ outputs ( $C_{\mathrm{CMAQ}}-C_{MMF}$ ). The histograms represent population-weighted concentrations.

This section aims to quantify the PM_2.5_ and O_3_ bias by different sources of CMAQ inputs. The apportioned biases of PM_2.5_ and O_3_ estimations from emission, boundary conditions, local meteorology, land-use data, and other unidentified factors and their spatial distributions are shown in Figure 4. Monthly biases from each component are listed in Supplementary Tables S4, S5, respectively. Compared with ML-MMF results, the CMAQ model underestimates PM_2.5_ for all regions by 0.99–4.56 μg/m^3^ (2–23%), where YCN is most underestimated (Figure 4a). The spatial distribution shows that the CMAQ model tends to overestimate (red) PM_2.5_ under hills and mountains and to underestimate (blue) in plains and basins, especially around coastal areas. The monthly patterns showed that April has more underestimations while October concentrations in western regions (CM, CT, YCN, and KP) are overestimated. Additionally, boundary conditions and local meteorology are the main driving forces to cause underestimation in January and April and overestimation in October. On the contrary, land-use data contributes a positive driving force in YCN, KP, and ET on the edge of hills or mountains, implying that the evaluation factors could cause positive biases when pollutants accumulate under hills or mountains. For O_3_ (Figure 4b), the CMAQ model overestimates for all regions by 5.13–10.96 ppb (17–29%), and O_3_ in almost western regions is overestimated. The monthly patterns showed July and October have more overestimations. NT, CM, and ET regions are more overestimated in July, while CT, YCN, and KP regions are more overestimated in October. Similar to PM_2.5_, boundary conditions and local meteorology are the main driving forces causing overestimation.

The results of the scenario design (Table 1) and bias quantification (Figure 4) show higher bias from boundary conditions, emphasizing the importance of boundary condition data quality for air quality modeling in Taiwan. The importance of boundary conditions results from frequent long-range transboundary air pollutants transported from mainland China in fall and winter (31, 32), which carry primary PM and precursors of secondary PM and O_3_ to Taiwan (27, 33), but such hourly- and daily-scale weather conditions and air pollutant concentrations are hard to be captured accurately by global or regional emission inventory and verified by ground-level observations. Additionally, the high sensitivity of the CMAQ model to boundary conditions suggested the need to improve aerosol mechanisms in the CMAQ model for a better simulation of particle matter transport and deposition over the marine boundary layer (34). For local meteorology, its inferior importance could be due to its collinearity with boundary conditions, or the current meteorological models still have limitations to predict over complex terrain and under extremely stable boundary layers (29, 35). On the other hand, emission inventory and land-use data only have relatively lower contributions, but it does not mean emission inventory and land-use data are not essential or not sensitive for CMAQ modeling. On the contrary, it reveals the emission and land-use data better explain the variance of PM_2.5_ and O_3_, so their derived biases are relatively lower than the bias from boundary conditions and local meteorology.

### 3.3 Further analysis of bias quantification

This section further utilizes the results of bias quantification and assesses the derived biases when different datasets are applied to calculate the number of premature deaths concerning PM_2.5_ and O_3_ exposure. Premature deaths estimated by using observations only, CMAQ output, ML-MMF output, and respective scenario outputs are shown in Table 2, and observation-derived premature deaths employed the observations from the closest monitoring stations. The improvement ratio (IR) of each scenario (S2-S5) calculates the improved estimation ratio compared with ML-MMF output (S1). Regional premature deaths are illustrated in Supplementary Table S6. Overall, compared with the ML-MMF estimations, using observations and uncorrected CMAQ output would overestimate deaths by 37 and 79%, respectively. The overestimations of using observations are because most monitoring stations are in populated areas that have higher air pollutant concentrations, thus air pollutant concentrations in suburban and rural areas would be overestimated. If using CMAQ-modeled estimations, most of the biased deaths are contributed by O_3_ exposure (171%, 6,518 deaths) other than PM_2.5_ exposure (−18%, −641 deaths). The scenario results (S2-S5) show similar impacts as the bias quantification (Table 2) shows, which including boundary conditions would contribute the most improvement (IR = 99%) for burden calculation, followed by local meteorology (IR = 91%), emphasizing the biases of acute BDs in Taiwan are much driven by transboundary pollution. Thus, to improve the calculation of acute BDs in Taiwan, the data quality of boundary conditions and local meteorology should be preferentially improved for local air quality and public health management.

**Table 2:** Estimated premature deaths due to daily PM_2.5_ and O_3_ exposure from closest observations, CMAQ, ML-MMF (S1), and individual scenario outputs [emissions (S2), boundary conditions (S3), local meteorology (S4), and land-use data (S5)].

Pollutant	Observation	CMAQ	ML-MMF (S1)	ML-MMF scenario (improvement ratio*)			
				Emis (S2)	BC (S3)	Met (S4)	LU (S5)
PM_2.5_	4,270	3,000	3,641	3,597 (93%)	3,636 (99%)	3,524 (82%)	3,082 (13%)
O_3_	5,948	10,331	3,813	2,902 (86%)	3,738 (99%)	3,419 (94%)	2,521 (80%)
Total	10,218	13,331	7,454	6,500 (84%)	7,374 (99%)	6,943 (91%)	5,603 (69%)

* $\mathrm{improvement ratio}=100\%-\frac{\left|Si-\mathrm{CMAQ}\right|}{\left|S1-\mathrm{CMAQ}\right|},i=2\cdots 5.$

Further sensitivity analysis estimates the premature deaths by using different PM_2.5_/O_3_ estimations and exposure risks. The premature deaths estimated by using observation/average-risk, observation/heterogeneous-risk, CMAQ/average-risk, CMAQ/heterogeneous-risk, ML-MMF/average-risk, and ML-MMF/heterogeneous-risk outputs are shown in Supplementary Figure S2 and Supplementary Table S7. For PM_2.5_, compared with the most ideal setting, ML-MMF/heterogeneous-risk deaths (*n* = 3,641), using closest observations would overestimate deaths by 17% (629) overall and 7–30% for western regions, and using CMAQ output would underestimate deaths by 18% (−641) overall. Furthermore, using average risk without considering heterogeneous risks would underestimate by 4% (−147) overall and 4–10% for respective regions. For O_3_, compared with the MMF/heterogeneous-risk deaths (*n* = 3,813), employing closest observations would overestimate total deaths by 56% (2135). Applying CMAQ output would highly overestimate total deaths by 171% (10,331 deaths), and the premature deaths in respective regions are overestimated by 114–303%. Additionally, using average risk would underestimate the deaths by 5% overall.

The results highlighted the potential BD biases by using different air pollutant concentration data and exposure risks. Both parameters can contribute considerable biases to BD calculation. Compared with the most ideal setting (ML-MMF output/heterogeneous risk), using observations only or CTM output only would contribute more bias than assuming averaged exposure risks for all areas. The higher sensitivity of air pollutant concentrations also implied that BD estimations using direct CTM output without observation-based correction or MMF would be potentially biased. Directly using observations for burden calculation also inferiorly reflects air quality for areas without monitoring stations.

## 4 Discussion

### 4.1 Perspectives

This study provides several perspectives for future regional air quality management and BD estimation. First, future air quality and risk management will need more precise and finer-scale air pollutant exposure estimations. Although present CTM applications provide long-term estimations for chronic exposure assessment, hourly or daily CTM estimations for short-term exposure assessment or acute BD calculation still need correction by observations. At this moment, our ML-MML framework is recommended for improving CTM performance and can serve as a post-processing procedure to improve model mechanisms and input data qualities. The bias quantification technique can provide a quantified bias structure of CTM estimations, so model developers can have priorities to optimize CTM algorithms, or users can have references to improve modeling input data quality in their study region. For example, in this study, the bias quantification of modeled-PM_2.5_ and O_3_ suggesting the data quality of boundary conditions and local meteorology should be first improved.

Furthermore, regional BD calculation should carefully assess the biases from different sources of air pollutant estimations and exposure risks. Both parameters are sensitive to burden calculation and could contribute to considerable biases. Either directly using CTM outputs without MMF or observations only could misrepresent the real exposure scenarios. Assuming a single exposure risk value for the population among different urbanization levels also overlooks the imbalance of exposure risks among urban, suburban, and rural areas. Spatially resolved exposure risks can employ local health data and be extracted through the developed framework in our previous study (23). In this study, using observations only (17% for PM_2.5_ and 56% for O_3_) or the uncorrected CTM estimations (−18% for PM_2.5_ and 171% for O_3_) contribute more biases to the premature deaths than employing averaged risks (−5% for PM_2.5_ and − 4% for O_3_), but this disparity could be region-specific and need to rely on local assessment.

### 4.2 Limitations

This study still has some limitations. First, the ML models highly depend on the number of monitoring stations to reflect the impact of geological characteristics around stations. In Taiwan, because most monitoring stations are located in coastal, basins, and plains, and there is very little monitoring data in mountainous areas to improve CTM performance, some ML techniques such as RF cannot properly utilize land-use variables for ML-MMF modeling. The other alternative source to obtain ground-level data is satellite data, but it still has some limitations. The satellite data may not provide hourly or daily-scale measurements, which is needed for acute disease burden calculations. Furthermore, the satellite data are still easily biased by clouds and columns of atmospheric layers. Second, although the bias quantification can quantify the bias from each modeling input, the bias of each component is still the combined bias of inaccuracy of inputs and imperfect mechanisms in the model, which cannot be easily differentiated. For example, the meteorology-contributed bias could be from the inaccurate estimations of meteorology modeling or imperfect physical/chemical mechanisms in the CTM.

## 5 Conclusion

More precise and finer-scale BD estimations are gradually recognized for regional air quality and risk management, but current regional BD estimations are still confounded by different sources of air pollutant concentration data and homogenous exposure risk among diverse urbanization levels. Although current ML applications can correct CTM results, the CTM mechanism and modeling input still hardly benefit from corrected results.

This study proposed an ML-MMF framework to improve regional BD estimation and further quantify the major bias sources of CTM estimations. In our case, bias quantification results showed that the CTM-modeled PM_2.5_/O_3_ are more affected by boundary conditions and local meteorology than other inputs, the derived premature deaths also presented that the acute BDs are mainly biased by boundary conditions (IR = 99%) and local meteorology (IR = 91%). Further sensitivity analysis highlighted the impact of different sources of air pollutant concentrations and exposure risks to BD estimations. Using observations only (17% for PM_2.5_ and 56% for O_3_) or the uncorrected CTM estimations (−18% for PM_2.5_ and 171% for O_3_) contribute more BD biases compared with employing averaged risk without considering urbanization levels (−5% for PM_2.5_ and − 4% for O_3_). However, the disparity could have regional specificity and need to rely on further regional assessment.

The study provides several perspectives for future regional air quality management and BD estimation. Since more air quality and health data become available, regional BD estimations should employ observation-corrected CTM results and finer-scale exposure risks. Furthermore, to improve CTM estimations, our bias quantification technique is recommended to provide a quantitative assessment of bias structure for improving input data quality and associated CTM mechanisms, so the study also provide references to improve CTM algorithms and modeling input data quality in their modeling domain.

## Data Availability

The original contributions presented in the study are included in the article/Supplementary material, further inquiries can be directed to the corresponding author.
