# Supplementary material for: Perspective improvement of regional air pollution burden of disease estimation by machine intelligence
Source: Front Public Health. 2025 Mar 12;13:1436838. doi: 10.3389/fpubh.2025.1436838 (PMC11937109; doi:10.3389/fpubh.2025.1436838)
Supplement: Supplementary file 1 [file Data_Sheet_1.pdf]

Table S1. Input variables selected for the ML-MMF framework.

Dataset	Source	Variables	
Observation	Monitoring stations	Daily $PM_{2.5}$ and MDA8 O_3	
Emission (n=24)	TEDS 10	$PM_{2.5}$, NOx, SOx, CO, NH_3, and VOCs emissions from point, mobile, area, and biomass sources.	
Boundary conditions (n=15)	WRF	air temperature (850 and 690 hPa), relative humidity (850 and 690 hPa), aerosol, NO_3, HNO_3, N_2O_5, NO, NO_2, sulfate, SO_2, VOCs, CO and O_3 at surface level	
Meteorological variables (n=21)	WRF	Surface (n=11)	surface pressure, PBL height, temperature at 2 m, mixing ratio at 2 m, wind speed, U wind component and V wind component, solar radiation reaching ground, precipitation, total cloud fraction, average liquid water content of cloud
		Pressure level (850 and 690 hPa) (n=10)	air temperature, potential vorticity, vertical velocity, U wind component and V wind component
Land-use (n=10)	CMAQ	evaluation, urban percent, dominant land use category based on MODIS (dummy variables)	

Table S2. Applied exposure risks for (a) daily $PM_{2.5}$ and (b) MDA8 O_3 among average, low, medium, and high urbanization levels.

**Exposure risks**		**Daily $PM_{2.5}$ (per 10 μg/m^3)**	**MDA8 O_3 (per 1 ppb)**
Average		1.25 (1.22-1.27)	1.06 (1.06-1.07)
Urbanization level	Low (rural)	1.31 (1.25-1.37)	1.07 (1.06-1.07)
	Medium (suburban)	1.14 (1.06-1.21)	1.06 (1.05-1.07)
	High (urban)	1.12 (1.05-1.19)	1.07 (1.06-1.08)

Table S3. Modeling performance of single model and multi-model (MM) approach for random forest (RF) and gradient boosted model (GBM).

Method	$PM_{2.5}$		O_3	
	**Train**	**Test**	**Train**	**Test**
KNN	0.82	0.82	0.79	0.80
RT	0.87	0.88	0.85	0.85
RF	0.93	0.93	0.92	0.92
GBM	0.93	0.93	0.91	0.90
CNN	0.98	0.91	0.96	0.88
MM-RF	0.99	0.89	0.98	0.85
MM-GBM	0.99	0.88	0.99	0.85

Table S4. Monthly average of population-weighted $PM_{2.5}$ concentration biases from emission data, boundary conditions, local meteorology, land-use data, and other unidentified factors.

Air quality region	Period	$PM_{2.5}$ Bias Source (μg/m^3)					
		Total	Emis	BC	Met	Landuse	Others
NT	4-month	-2.25	1.11	-1.50	-1.07	-0.66	-0.21
	Jan	-2.68	1.38	-2.59	-1.48	-0.64	0.44
	Apr	-6.98	1.14	-4.16	-3.13	-0.45	-0.55
	Jul	1.46	0.77	1.17	0.64	-0.80	-0.32
	Oct	-0.95	1.19	-0.73	-0.37	-0.76	-0.42
CM	4-month	-2.31	0.94	-1.91	-1.45	-0.30	0.25
	Jan	-1.60	1.18	-2.07	-1.15	-0.28	0.63
	Apr	-8.82	1.43	-5.91	-4.16	-0.36	-0.24
	Jul	-1.17	0.84	-0.81	-1.20	-0.52	0.40
	Oct	2.14	0.30	0.98	0.63	-0.05	0.23
CT	4-month	-2.99	0.77	-2.00	-1.40	-0.24	-0.21
	Jan	-2.88	1.30	-3.02	-1.13	-0.28	0.07
	Apr	-10.45	1.25	-6.58	-4.29	-0.33	-0.60
	Jul	-1.76	0.82	-1.04	-0.94	-0.44	-0.16
	Oct	2.91	-0.30	2.59	0.74	0.11	-0.13
YCN	4-month	-4.56	-1.15	-2.71	-1.78	0.93	-0.02
	Jan	-6.36	-1.52	-3.31	-2.71	0.99	-0.31
	Apr	-10.89	-1.48	-6.88	-3.99	1.11	0.36
	Jul	-2.65	-0.85	-1.99	-1.13	1.27	-0.08
	Oct	1.46	-0.75	1.36	0.57	0.39	-0.06
KP	4-month	-0.99	-0.50	-0.77	-0.62	0.52	0.34
	Jan	-2.23	-0.62	-0.67	-1.63	0.15	0.43
	Apr	-6.18	-0.70	-4.83	-2.86	1.03	0.82
	Jul	0.22	-0.69	-0.41	-0.21	1.58	0.18
	Oct	4.09	0.01	2.61	2.11	-0.44	-0.05
ET	4-month	-3.72	0.77	-4.30	-1.37	5.60	-5.03
	Jan	-4.53	0.87	-4.98	-1.73	5.59	-5.23
	Apr	-7.15	0.81	-7.49	-2.08	5.87	-4.41
	Jul	-1.45	0.61	-2.02	-0.70	5.29	-5.23
	Oct	-1.86	0.77	-2.10	-0.99	5.26	-5.95

Table S5. Monthly average of population-weighted O_3 concentration biases from emission data, boundary conditions, local meteorology, land-use data, and other unidentified factors.

Air quality region	Period	O_3 Bias Source (ppb)					
		Total	Emis	BC	Met	Landuse	Others
NT	4-month	10.96	-1.71	7.47	5.06	0.74	-0.17
	Jan	3.30	-0.38	1.92	1.43	0.22	0.27
	Apr	8.56	-1.91	5.80	4.65	0.89	-0.23
	Jul	23.10	-3.05	15.28	9.91	1.23	-0.27
	Oct	8.79	-1.43	6.33	4.06	0.59	-0.47
CM	4-month	7.99	0.90	5.61	3.92	-1.88	-0.26
	Jan	2.50	0.14	2.14	1.44	-0.71	-0.33
	Apr	4.81	0.84	3.22	2.99	-2.02	-0.06
	Jul	14.62	1.36	9.47	6.35	-2.28	-0.28
	Oct	9.90	1.25	7.17	4.69	-2.51	-0.34
CT	4-month	9.81	-0.28	7.28	3.33	-0.38	0.18
	Jan	4.94	-0.10	3.77	1.71	-0.17	0.20
	Apr	4.85	-0.24	3.91	1.67	-0.37	0.06
	Jul	13.66	-0.33	9.73	4.31	-0.37	0.31
	Oct	15.65	-0.44	11.28	5.27	-0.60	0.13
YCN	4-month	8.40	1.70	6.39	2.76	-1.66	-0.61
	Jan	4.59	0.82	4.56	1.36	-0.94	-0.96
	Apr	1.87	1.37	2.25	0.60	-1.50	-0.77
	Jul	12.15	1.96	8.23	3.92	-1.41	-0.55
	Oct	14.77	2.58	10.16	4.98	-2.75	-0.20
KP	4-month	7.21	-1.24	5.74	3.60	0.43	-0.59
	Jan	7.36	-0.98	6.24	3.38	0.34	-1.22
	Apr	-1.70	-0.86	-1.35	-0.14	0.32	-0.50
	Jul	10.69	-1.22	7.50	5.00	0.34	-0.15
	Oct	12.20	-1.83	8.72	5.65	0.71	-0.50
ET	4-month	5.13	2.28	4.03	2.47	-2.02	-1.27
	Jan	3.16	1.24	3.04	1.81	-1.45	-1.22
	Apr	1.62	2.72	1.15	2.01	-2.69	-1.39
	Jul	12.48	3.62	8.49	4.26	-2.59	-1.30
	Oct	3.14	1.45	2.94	1.54	-1.30	-1.18

Table S6. Premature deaths due to daily $PM_{2.5}$ and O_3 exposure from closest observations, CMAQ, ML-MMF (S1), and individual scenario outputs (emissions (S2), boundary conditions (S3), local meteorology (S4), and land-use data (S5)) for six air quality regions.

Pollutant/ Region	Observation	CMAQ	ML-MMF (S1)	ML-MMF scenario			
				Emis (S2)	BC (S3)	Met (S4)	LU (S5)
**$PM_{2.5}$**							
NT	1042	747	925	725	950	837	706
CM	242	193	206	231	228	202	174
CT	885	587	742	688	738	753	613
YCN	1168	575	891	997	833	857	738
KP	912	892	853	942	852	864	826
ET	21	5	24	15	36	10	26
**O_3**							
NT	1542	4011	995	1069	1015	839	917
CM	371	544	225	144	233	194	125
CT	1299	2124	941	536	919	935	500
YCN	1349	1738	812	510	798	711	474
KP	1330	1823	810	635	734	732	489
ET	58	91	29	7	39	6	17
**$PM_{2.5} + O_3$**							
NT	2584	4758	1921	1794	1964	1676	1622
CM	613	737	431	375	462	396	299
CT	2184	2711	1682	1225	1657	1688	1112
YCN	2516	2313	1703	1507	1631	1568	1212
KP	2242	2715	1664	1577	1585	1597	1315
ET	80	97	53	22	75	17	43
Total	10218	13331	7454	6500	7374	6943	5603

Table S7. Premature deaths for six air quality regions calculated from observation/average-risk, observation/heterogeneous-risk, CMAQ/average-risk, CMAQ/heterogeneous-risk, MMF/average-risk, and MMF/heterogeneous-risk outputs due to (a) daily $PM_{2.5}$ and (b) MDA8 O_3 exposure.

**Air quality region**	**Premature deaths #<br>(different deaths, ratio compared with MMF/Heterogeneous risk-100%)**					
	**Obs/Average risk**	**Obs/ Heterogeneous risk**	**CMAQ/ Average risk**	**CMAQ/ Heterogeneous risk**	**MMF/ Average risk**	**MMF/ Heterogeneous risk**
**(a) $PM_{2.5}$**						
NT	970<br>(45, 5%)	1042<br>(117, 13%)	712<br>(-213, -23%)	747<br>(-178, -19%)	856<br>(-69, -7%)	925<br>(0, 0%)
CM	219<br>(13, 6%)	242<br>(36, 18%)	182<br>(-24, -12%)	193<br>(-13, -6%)	189<br>(-17, -8%)	206<br>(0, 0%)
CT	934<br>(192, 26%)	885<br>(143, 19%)	671<br>(-71, -10%)	587<br>(-154, -21%)	804<br>(63, 8%)	742<br>(0, 0%)
YCN	1062<br>(172, 19%)	1168<br>(277, 31%)	522<br>(-368, -41%)	575<br>(-316, -35%)	803<br>(-87, -10%)	891<br>(0, 0%)
KP	890<br>(37, 4%)	912<br>(59, 7%)	863<br>(10, 1%)	892<br>(39, 5%)	819<br>(-34, -4%)	853<br>(0, 0%)
ET	18<br>(-6, -24%)	21<br>(-3, -11%)	5<br>(-19, -78%)	5<br>(-19, -78%)	23<br>(-2, -7%)	24<br>(0, 0%)
Total	4093<br>(452, 12%)	4270<br>(629, 17%)	2955<br>(-686, -19%)	3000<br>(-641, -18%)	3494<br>(-147, -4%)	3641<br>(0, 0%)
**(b) O_3**						
NT	1100<br>(105, 10%)	1542<br>(546, 55%)	3832<br>(2837, 285%)	4011<br>(3016, 303%)	930<br>(-66, -7%)	995<br>(0, 0%)
CM	245<br>(20, 9%)	371<br>(145, 65%)	532<br>(307, 136%)	544<br>(318, 141%)	220<br>(-5, -2%)	225<br>(0, 0%)
CT	999<br>(59, 6%)	1299<br>(358, 38%)	2053<br>(1112, 118%)	2124<br>(1183, 126%)	889<br>(-52, -6%)	941<br>(0, 0%)
YCN	863<br>(50, 6%)	1349<br>(536, 66%)	1697<br>(885, 109%)	1738<br>(926, 114%)	788<br>(-25, -3%)	812<br>(0, 0%)
KP	934<br>(124, 15%)	1330<br>(519, 64%)	1763<br>(953, 118%)	1823<br>(1012, 125%)	771<br>(-39, -5%)	810<br>(0, 0%)
ET	35<br>(7, 23%)	58<br>(30, 104%)	88<br>(60, 209%)	91<br>(63, 219%)	28<br>(-1, -4%)	29<br>(0, 0%)
Total	4176<br>(364, 10%)	5948<br>(2135, 56%)	9966<br>(6153, 161%)	10331<br>(6518, 171%)	3624<br>(-189, -5%)	3813<br>(0, 0%)

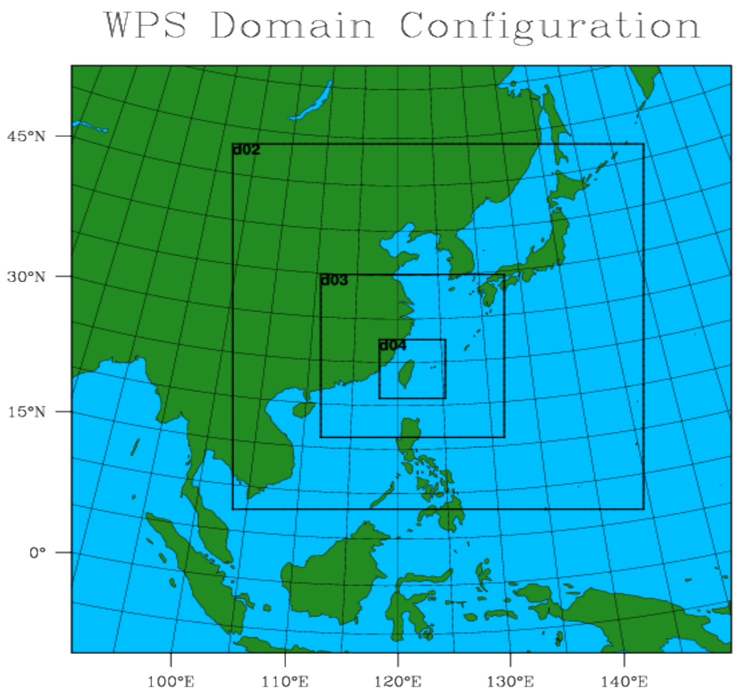

Figure S1. Overview of the simulated 4 nested domains from domain 1 (81 km) to domain 4 (3 km)

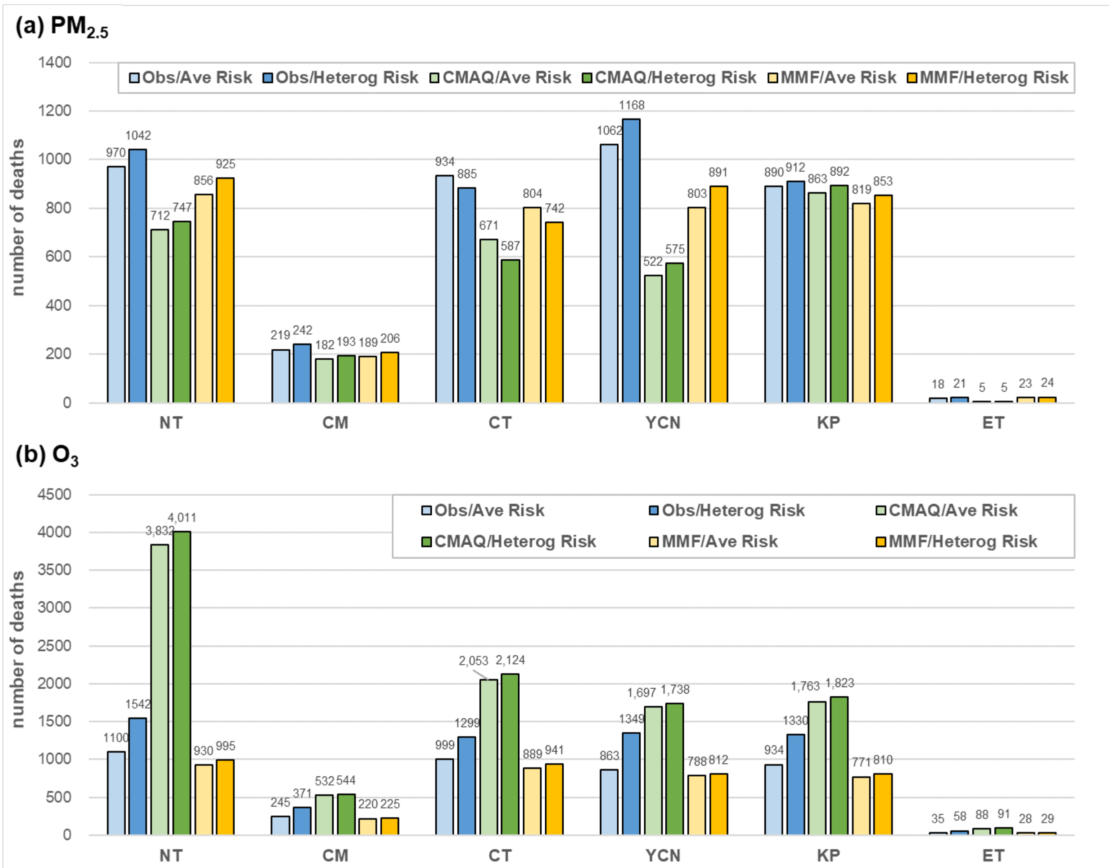

Figure S2. Premature deaths for six air quality regions calculated from observation/average-risk, observation/heterogeneous-risk, CMAQ/average-risk, CMAQ/heterogeneous-risk, MMF/average-risk, and MMF/heterogeneous-risk outputs due to (a) daily $PM_{2.5}$ and (b) MDA8 O_3 exposure.

Appendix I. Scenario Design and Testing for Measurement-Model Fusion (ML-MMF)

Methods

Different scenarios were designed to quantify bias between CMAQ raw output and observations (Table S8). S1_BASE is the base model that uses all inputs for prediction and serves as a baseline for comparing with the other scenarios, and the following scenarios can be classified into two categories: including scenarios (S2_EM, S3_BC, S4_MT, S5_LU) and excluding scenarios (S6_nEM, S7_nBC, S8_nMT, S9_nLU). Compared with CMAQ output, including scenarios assess the individual improved performance by including each data (emission, boundary condition, meteorological, and land-use data) and illustrate individual capability for MMF. Additionally, compared with S1_BASE, excluding scenarios exclude each data individually and show the decreased performance due to the lack of individual data.

The bias quantification technique utilized $PM_{2.5}$ and O_3 estimations from each scenario. For each air quality region, total bias (ΔC_{Total}) was defined by the changing population-weighted $PM_{2.5}$ and O_3 between CMAQ raw output and S1_BASE ($\Delta C_{Total} = C_{CMAQ} - C_{S1\_BASE}$). The modeling capability of each component (emission, boundary condition, meteorology, and land-use data) was defined by either the including scenarios or the excluding scenarios. For example, the modeling capability of emissions was defined by the changing concentration between CMAQ output and S2_EM ($\Delta C_{1,EM} = C_{CMAQ} - C_{S2\_EM}$) or the changing concentration between S1_BASE and S6_nEM ($\Delta C_{2,EM} = C_{S1\_BASE} - C_{S6\_nEM}$). For all components, the calculated biases were further used to apportion their contributions to the total bias through multiple linear regression (MLR):

$$\Delta C_{Total} = \beta_0 + \beta_1 \Delta C_{i,EM} + \beta_2 \Delta C_{i,BC} + \beta_3 \Delta C_{i,MT} + \beta_4 \Delta C_{i,LU} + \varepsilon$$

where i represent the application of including scenarios ($\Delta C_{1,EM}$, $\Delta C_{1,BC}$, $\Delta C_{1,MT}$, and $\Delta C_{1,LU}$) or excluding scenarios ($\Delta C_{2,EM}$, $\Delta C_{2,BC}$, $\Delta C_{2,MT}$, and $\Delta C_{2,LU}$); β_0 is the intercept; β_1 to β_4 represents contributed bias with a unit increase of delta $PM_{2.5}$ or O_3 concentration. The products including $\beta_1 \Delta C_{i,EM}$, $\beta_2 \Delta C_{i,BC}$, $\beta_3 \Delta C_{i,MT}$, and $\beta_4 \Delta C_{i,LU}$ are the changed concentrations from emissions, boundary conditions, meteorology, and land-use data, respectively. ε are residuals and represent biases from other unidentified factors.

The results of MLR models showed that using including scenarios (R^2=0.96 for $PM_{2.5}$; R^2=0.98 for O_3) had a higher explained variance compared with using excluding scenarios (R^2=0.44 for $PM_{2.5}$; R^2=0.32 for O_3), so the further bias quantification analysis was based on including scenarios.

Table S8. Modeling performance evaluation (R^2) of $PM_{2.5}$ and O_3 for different ML techniques and scenarios

Scenario	Input data	KNN		RT		RF		GBM		CNN	
		Train	Test	Train	Test	Train	Test	Train	Test	Train	Test
**$PM_{2.5}$**											
S1_BASE	CMAQ + Emis + BC + Met+ LU	0.68	0.68	0.76	0.77	0.87	0.87	0.86	0.86	0.95	0.83
S2_EM	CMAQ + Emis	0.51	0.54	0.47	0.48	0.59	0.59	0.58	0.59	0.62	0.57
S3_BC	CMAQ + BC	0.75	0.76	0.74	0.74	0.77	0.77	0.76	0.77	0.79	0.77
S4_MT	CMAQ + Met	0.71	0.73	0.63	0.65	0.81	0.82	0.76	0.77	0.84	0.68
S5_LU	CMAQ + LU	0.48	0.49	0.46	0.48	0.50	0.51	0.51	0.52	0.52	0.49
S6_nEM	CMAQ + BC + Met + LU	0.72	0.74	0.77	0.78	0.87	0.87	0.85	0.85	0.94	0.82
S7_nBC	CMAQ + Emis + Met + LU	0.53	0.54	0.62	0.67	0.80	0.81	0.79	0.79	0.91	0.69
S8_nMT	CMAQ + Emis + BC + LU	0.64	0.65	0.74	0.72	0.83	0.83	0.83	0.83	0.93	0.84
S9_nLU	CMAQ + Emis + BC + Met	0.70	0.70	0.77	0.77	0.87	0.87	0.86	0.86	0.94	0.83
**O_3**											
S1_BASE	CMAQ + Emis + BC + Met+ LU	0.62	0.63	0.73	0.73	0.84	0.85	0.82	0.81	0.93	0.78
S2_EM	CMAQ + Emis	0.51	0.49	0.49	0.48	0.53	0.52	0.56	0.55	0.58	0.51
S3_BC	CMAQ + BC	0.78	0.77	0.75	0.74	0.78	0.78	0.77	0.77	0.79	0.76
S4_MT	CMAQ + Met	0.70	0.69	0.62	0.61	0.78	0.79	0.74	0.73	0.78	0.63
S5_LU	CMAQ + LU	0.51	0.47	0.49	0.48	0.52	0.49	0.53	0.51	0.54	0.50
S6_nEM	CMAQ + BC + Met + LU	0.71	0.71	0.72	0.74	0.84	0.85	0.81	0.81	0.90	0.79
S7_nBC	CMAQ + Emis + Met + LU	0.51	0.50	0.61	0.60	0.77	0.77	0.75	0.74	0.85	0.66
S8_nMT	CMAQ + Emis + BC + LU	0.59	0.60	0.73	0.73	0.81	0.81	0.79	0.79	0.88	0.78
S9_nLU	CMAQ + Emis + BC + Met	0.67	0.68	0.71	0.72	0.84	0.85	0.82	0.81	0.89	0.78
